# Initial Investigation of Vericiguat Efficacy in the Treatment of Recurrently Hospitalised Heart Failure Patients

**DOI:** 10.7759/cureus.91192

**Published:** 2025-08-28

**Authors:** Aleksandrs Vasiljevs, Karlis Trusinskis

**Affiliations:** 1 Department of Internal Diseases and Pathology, Riga Stradins University, Riga, LVA; 2 Latvian Center of Cardiology, Pauls Stradiņš Clinical University Hospital, Riga, LVA; 3 Faculty of Medicine and Life Sciences, University of Latvia, Riga, LVA

**Keywords:** advanced heart failure, heart failure, hypotension, reduced ejection fraction, vericiguat

## Abstract

Introduction: Heart failure is a global health problem that has led to an increase in hospitalisations, a decrease in quality of life and an increase in premature death. Rehospitalisation due to worsening heart failure is an indicator of poor prognosis and is directly correlated with life expectancy. Furthermore, additional hospitalisations increase the financial burden on medical systems.

Objective: The objective of this study was to evaluate the effectiveness of the novel heart failure medication vericiguat in reducing hospitalisations and improving the clinical parameters of patients with heart failure with a reduced ejection fraction who are hospitalised with heart failure decompensation and need intravenous diuretics for decongestion. Additionally, reassure practitioners in vericiguat safety and efficiency to increase the number of patients receiving novel medications.

Methods: We conducted a retrospective observational study in patients with decompensated heart failure who were hospitalised at the Latvian Center of Cardiology. Data were collected for the period between November 2023 and November 2024, using patients' medical documentation. Vericiguat was initiated by physicians accordingly to the European guidelines on treating advanced heart failure. The aim of the study was to evaluate the effect of adding a novel medication on patients' hospitalisation rates and changes in clinical parameters. Clinical, echocardiographic and laboratory parameters were assessed during index hospitalisation and after one year with optimally tolerated medical therapy that included vericiguat. Statistical analysis was performed using SPSS version 22 (IBM Corp., Armonk, New York, USA).

Results: In our study, more than half of the heart failure patients who received optimal medical treatment that included vericiguat were not rehospitalised and their condition did not worsen within 12 months of follow-up. With the optimisation of medical therapy and the addition of vericiguat, there was a statistically significant increase in the left ventricular ejection fraction and a statistically significant decrease in NT-proBNP levels.

Conclusions: Vericiguat can be added for the treatment of patients with a reduced ejection fraction and episodes of decompensation, showing promising results in decreasing additional hospitalisations and improving the clinical outcomes of patients.

## Introduction

Heart failure has become a global health problem, leading to increased rates of hospitalisation, decreased quality of life, and increased health care system expenses [1]. Heart failure is a complication of multiple conditions, such as coronary heart disease, hypertension, and valvular diseases, and is a complication of chemotherapy and radiotherapy. Heart failure affected 64.3 million people worldwide in 2017. Its prevalence is expected to increase due to improved survival following heart failure diagnosis and the overall longer life expectancy of the general population [2]. Additionally, the prevalence of risk factors such as ischaemic heart disease, hypertensive heart disease, and cardiomyopathy/myocarditis has increased [3]. Advanced heart failure patients have high rates of mortality and morbidity. Previous trials have shown that three-year survival in patients with heart failure and a reduced ejection fraction is approximately 30% [4]. Patients with advanced heart failure have a one-year mortality rate of 49.9% [4].

Patients with advanced heart failure tend to have hypotension, which is a limiting factor for the optimisation of medical therapy [5]. Considering that recommended heart failure therapy tends to lower patients' blood pressure, it becomes an obstacle for achieving the quadruple therapy recommended by the guidelines, and patients tolerate suboptimal dosages or must limit their use of certain medications, decreasing the efficiency of treatment. Comorbidities, such as kidney failure, diabetes and arrhythmia, also increase the risk of a poor long-term prognosis [6].

Vericiguat is a direct soluble guanylyl cyclase stimulator that works synergistically with and independently of NO to increase intracellular cGMP production; by increasing intracellular cGMP levels, vericiguat has been shown to promote vasodilation and smooth muscle relaxation with minimal or no decrease in blood pressure [7].

The European Society of Cardiology guidelines with IIb recommendations suggest that vericiguat may be considered for patients with New York Heart Association (NYHA) class II-IV disease who have worsening heart failure despite treatment with optimal therapy to reduce the risks of cardiovascular-associated mortality and heart failure-associated hospitalisation [8].

The objective of this study was to evaluate the effectiveness of vericiguat in reducing hospitalisations and improving left ventricular ejection fraction and NT-proBNP. By presenting new data, we hope to improve clinical practices and implement novel treatment options.

## Materials and methods

Study population

We conducted a retrospective observational study in patients hospitalised with heart failure decompensation who received intravenous diuretics for congestion therapy at Pauls Stradiņš University Clinical Hospital for the period of index hospitalisation between November 2023 and November 2024, and data were collected at index hospitalisation and in one year from hospitalisation. Patients were hospitalised in Pauls Stradiņš University Clinical Hospital, which is an advanced medical centre allowing complete patient examination and other specialities consultations, providing complete care for patients with complicated conditions. Patients' medical records were used to obtain clinical information. Initiation of vericiguat was based on the treating physician's decision, based on clinical guidelines on heart failure treatment. No randomisation was done; the decision was based on the clinical parameters of patients. Patients' clinical parameters, echocardiography data, and natriuretic peptide levels were collected at the time of index hospitalisation and at the 12-month follow-up. Echocardiography was performed in one centre using the same protocol to minimise observer variability. One year after the index hospitalisation, the rates of rehospitalisation due to heart failure were recorded using hospital records.

Inclusion and exclusion criteria

Hospitalised patients with worsening heart failure and a reduced ejection fraction of less than 45% who had been hospitalised due to worsening heart failure and had received intravenous diuretic therapy were included in the study, similarly to the initial vericiguat VICTORIA trial [9]. Vericiguat was initiated when patients stopped receiving intravenous diuretics. Vericiguat was initiated when standard therapy was titrated to maximally tolerated dosages and medication groups. Up titration was often limited by hypotension.

Exclusion criteria were glomerular filtration rate less than 15 mL/min/1.73 m^2^ or chronic dialysis, severe hepatic insufficiency and concurrent use or anticipated use of phosphodiesterase type 5 (PDE5) inhibitors.

Ethical considerations

Institutional review board (IRB) approval for the study titled "Initial experience of Vericiguat treatment in Latvia" was obtained from the IRB under reference number 2-PĒK-4/735/2025. The title was slightly modified after review and English language editing to improve language clarity and better represent the patients' group. Written informed consent was waived because of the retrospective study design. All the data were fully anonymised.

Sample size

Due to the novelty of vericiguat in clinical practice, our centre was the first in Latvia to incorporate vericiguat into standard quadruple heart failure therapy. Consequently, the number of patients is limited to 18, as this initial patient group enabled us to form our opinion and implement this novel medication in clinical practice. Subgroups were not analysed due to the small sample size of patients.

Statistical analysis

Statistical analysis was performed using SPSS version 22 (IBM Corp., Armonk, New York, USA). Given the small sample size (n=18), descriptive statistics were used. The Wilcoxon signed-rank test was used to calculate changes in NT-proBNP and ejection fraction.

## Results

In our study, 18 patients were enrolled: 3 (16.7%) women and 15 (83.3%) men. Patient age ranged from 41 to 89 years, with a median of 65 years. The comorbidities at the time of index hospitalisation were as follows: atrial fibrillation 10 (55.6%), coronary heart disease 9 (50%), history of myocardial infarction 6 (33.3%), chronic kidney disease 7 (38.9%), and hypotension, defined as a systolic blood pressure less than 95 mm HG 9 (50%) and diabetes 2 (11.1%) (Table 1). Arterial hypotension 9 (50%) had the greatest impact on therapy limitations. Most of the patients 16 (77.7%) had class III of heart failure based on New York Heart Association Functional Classification, 2 (11.1%) had class II, and 2 (11.1%) had class IV heart failure (Table 1). 

**Table 1:** Patient characteristics.

Patient characteristics	N (%)
Age	41-89
Women	3 (16.7%)
Men	15 (83.3%)
Comorbidities	
Atrial fibrillation	10 (55.6%)
Coronary heart disease	9 (50%)
History of myocardial infarction	6 (33.3%)
Chronic kidney disease	7 (38.9%)
Diabetes	2 (11.1%)
Arterial hypotension	9 (50%)
Class of heart failure (New York Heart Association)	
II class	2 (11.1%)
III class	16 (77.7%)
IV class	2 (11.1%)

Before index hospitalisation, all patients received β-blockers, 15 (83.3%) received mineralocorticoid receptor antagonists, 12 (66.6%) received sodium-glucose co-transporter 2 inhibitors, 4 (22.2%) received sacubitril/valsartan, and 4 (22.2%) received angiotensin-converting enzyme (ACE) inhibitors.

**Figure 1:**
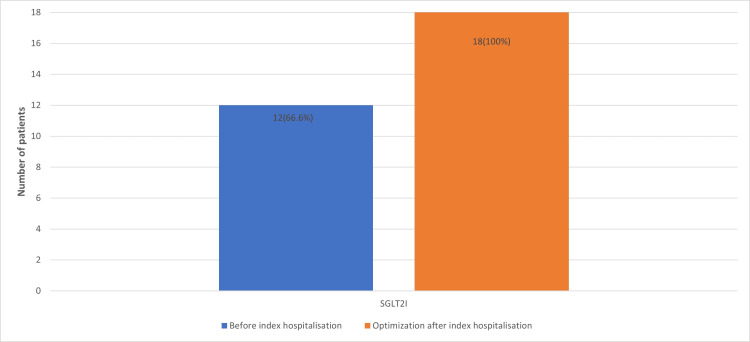
Number of patients receiving sodium-glucose transport protein 2 (SGLT2) inhibitors before index hospitalisation and after therapy optimisation.

**Figure 2:**
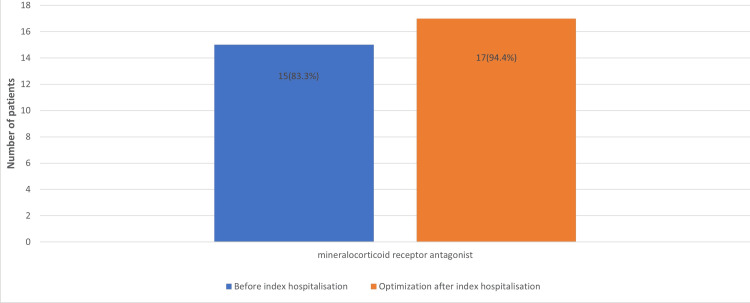
Number of patients receiving mineralocorticoid receptor antagonists before index hospitalisation and after therapy optimisation.

**Figure 3:**
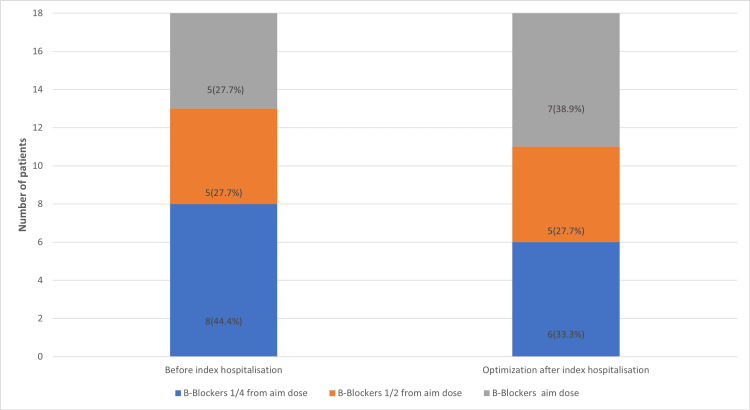
Number of patients and dosages of β-blockers by the patients before index hospitalisation and after therapy optimisation.

**Figure 4:**
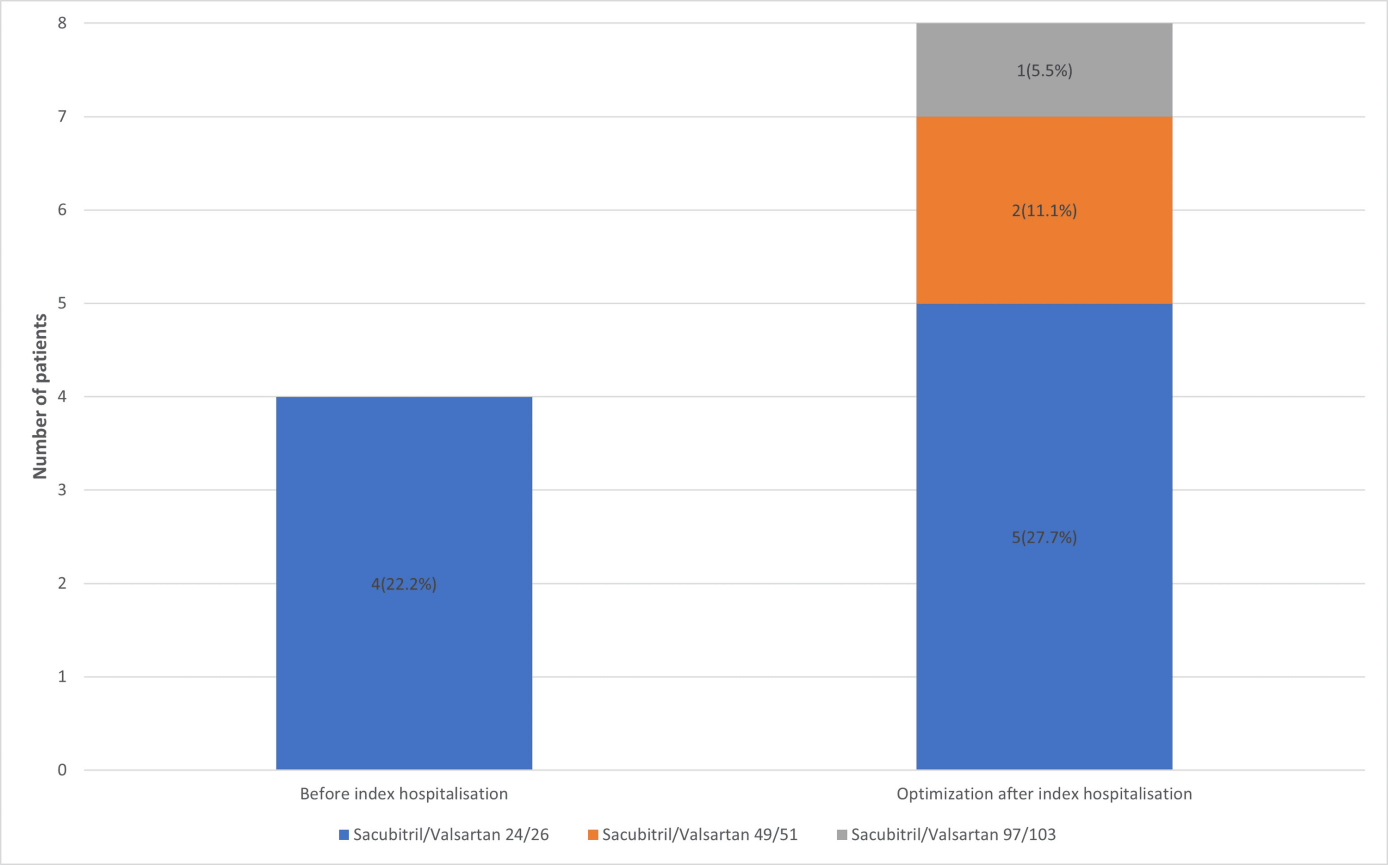
Number of patients and dosages of sacubitril/valsartan received by the patients before index hospitalisation and after therapy optimisation.

At index hospitalisation, patients received decongestion therapy with intravenous diuretics, and after clinical improvement, therapy was optimised according to the guidelines, and vericiguat was added. When sodium-glucose co-transporter-2 (SGLT2) inhibitor usage increased to 18 (100%) (Figure 1), mineralocorticoid receptor antagonist usage increased to 17 (94.4%) (Figure 2), and patients also better tolerated higher dosages of β-blockers (Figure 3), sacubitril/valsartan (Figure 4) and angiotensin converting enzyme inhibitors.

At index hospitalisation, when intravenous diuretics were stopped, patients received vericiguat at a dose of 2.5 mg. The optimal dose was titrated ambulatory and was found to be 10 mg. All patients tolerated this dose; patients did not have any adverse events using vericiguat.

**Figure 5:**
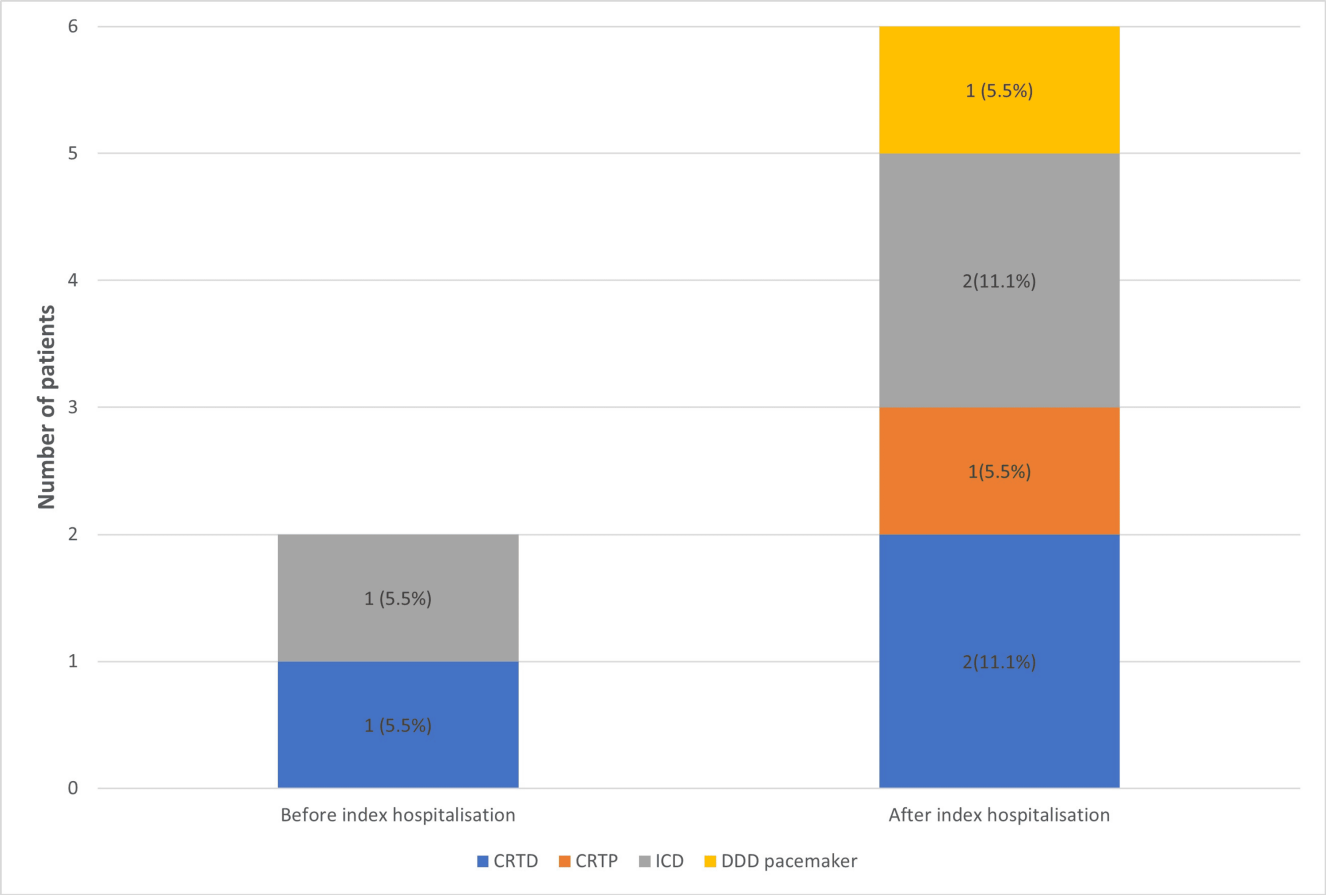
Device therapy before and after index hospitalisation. CRTD: cardiac device with resynchronisation therapy and defibrillator functions, CRTP: cardiac device with resynchronisation therapy, ICD: implantable cardioverter-defibrillator, DDD: dual-chamber pacemaker.

Before the index hospitalisation, 1 (5.5%) patient had an implanted implantable cardioverter-defibrillator (ICD), and 1 (5.5%) patient had an implanted cardiac device with resynchronisation therapy and defibrillator functions (CRTD). After the index hospitalisation, 2 (11.1%) ICD, 1 (5.5%) dual chamber pacemaker, 1 (5.5%) CRTD, and 1 (5.5%) cardiac device with resynchronisation therapy (CRTP) were implanted (Figure 5). Decisions about implanting a device were based on guideline recommendations.

**Figure 6:**
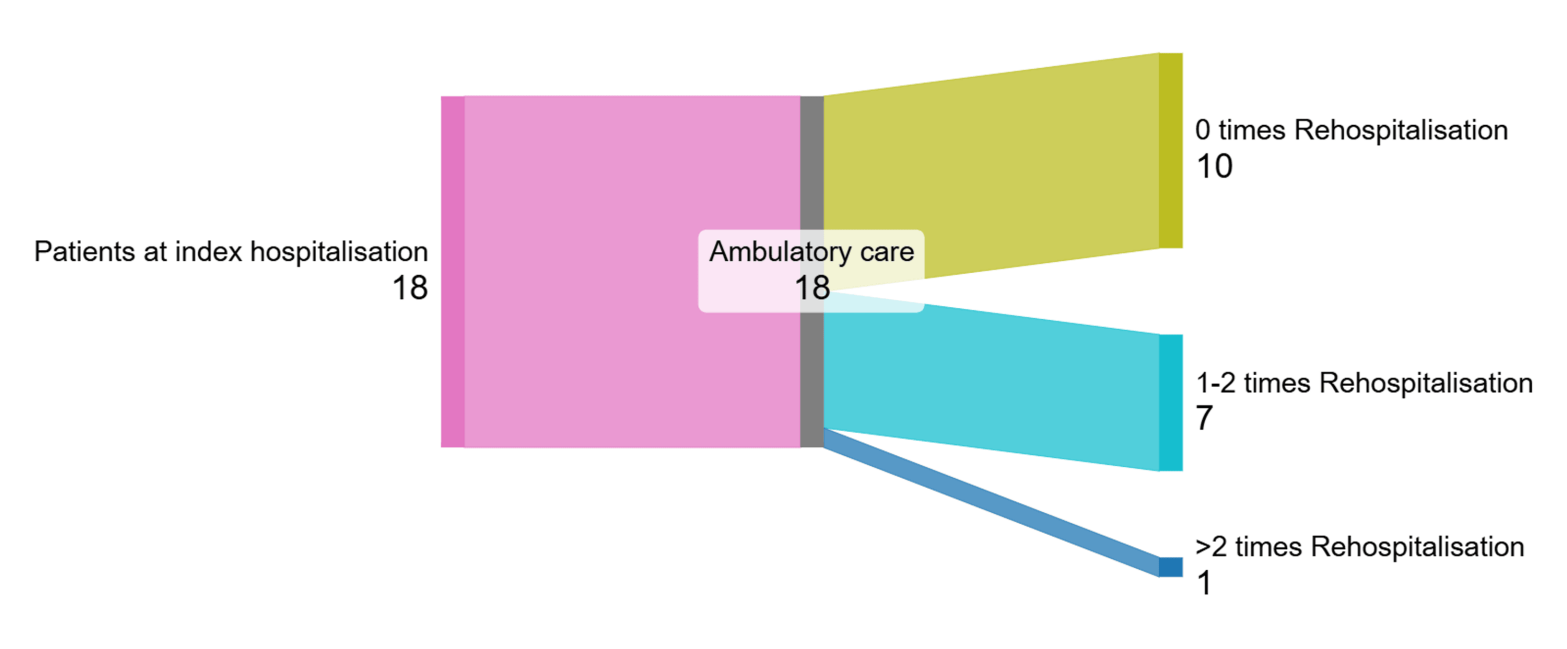
Rehospitalisation one year after the index hospitalisation due to heart failure decompensation.

The number of rehospitalisations due to heart failure decompensation after one year of follow-up after the index hospitalisation is shown in Figure 6. 10 (55.6%) patients were not hospitalised due to heart failure decompensation, 7 (38.9%) patients were hospitalised one to two times, and 1 (5.5%) patient was hospitalised more than two times. Two patients had symptoms of heart failure decompensation and increased oedema, which was managed by adjusting loop diuretic doses out of hospital; patients were included in the not rehospitalised group.

**Figure 7:**
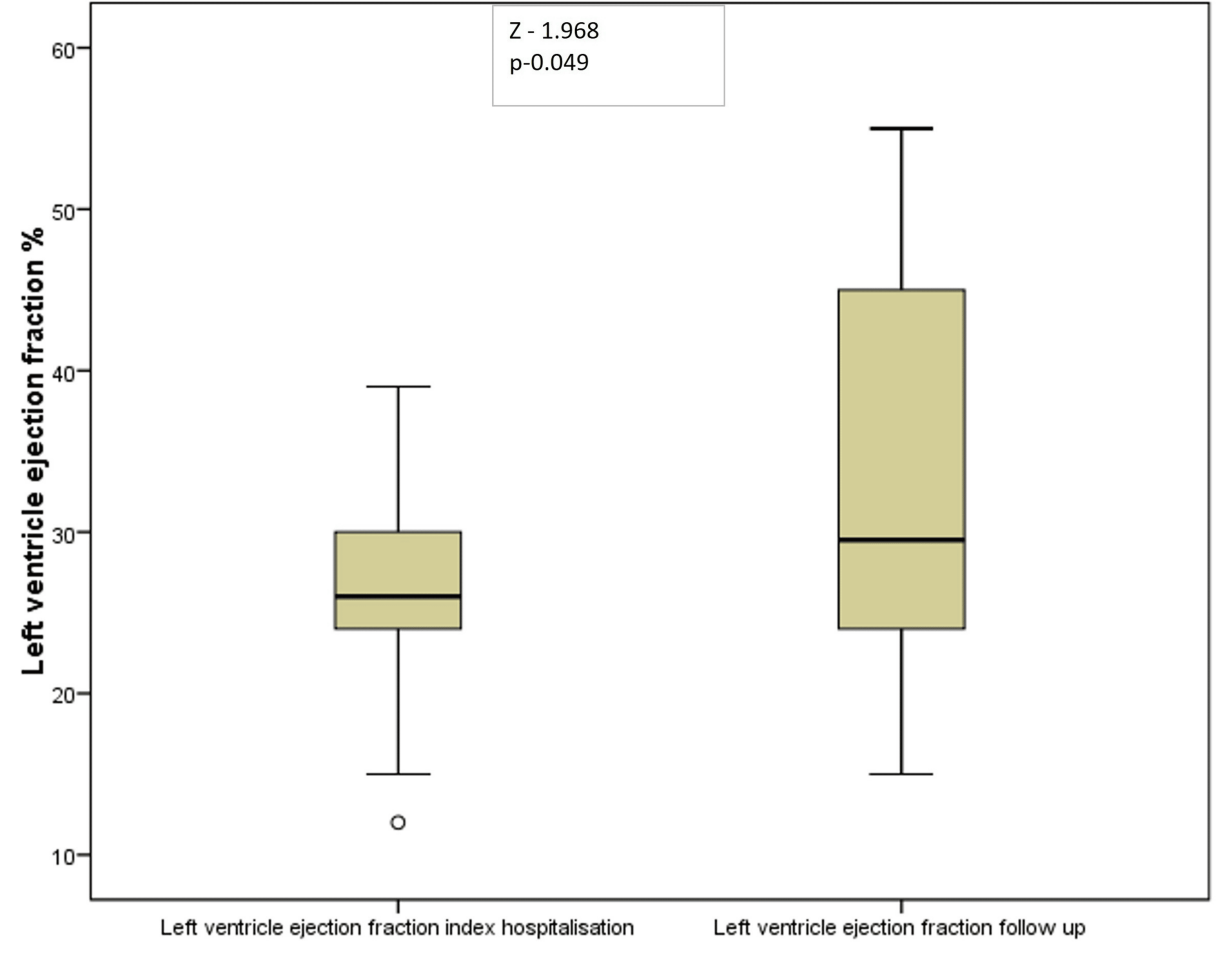
Left ventricular ejection changes at the one-year follow-up.

The average ejection fraction rate on admission was 27%. After therapy at the one-year follow-up, the average ejection fraction increased to 33%. The average ejection fraction increased significantly by 22% on average (p=0.049) (Figure 7).

**Figure 8:**
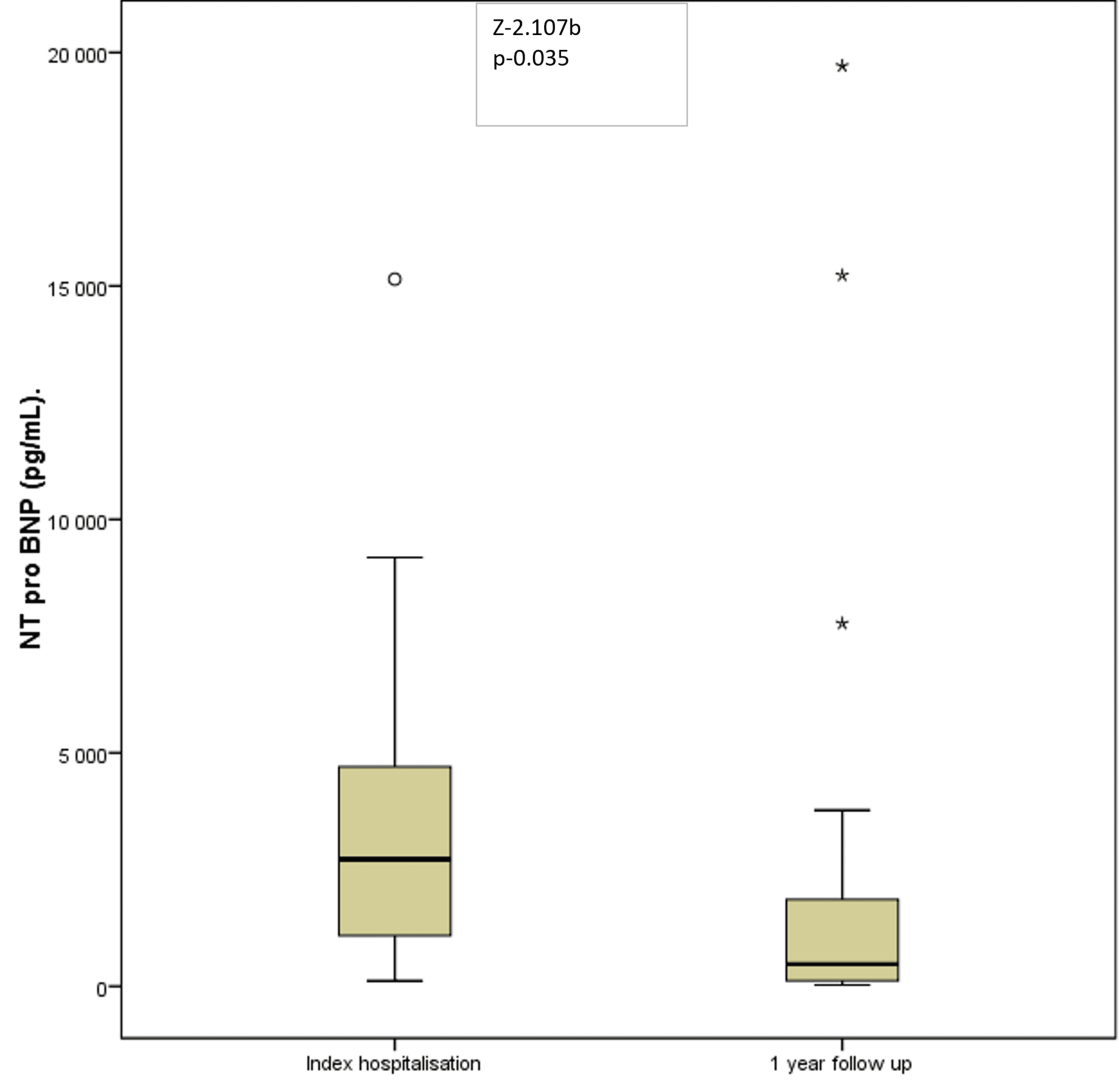
NT-proBNP changes (pg/mL) at the one-year follow-up.

The average NT-proBNP level on admission was 5642 pg/ml, and at one year, the average NT-proBNP level was 3117 pg/ml. The average decrease was 44.7%, which was statistically significant (p=0.035) (Figure 8).

All patients lived one year after the index hospitalisation and reached one year follow-up, and all patients used vericiguat as prescribed.

## Discussion

The main finding of the study is the reduced rate of rehospitalisation due to heart failure decompensation in the studied patient group that was administered vericiguat. In a recent SGLT2 inhibitor era trial with 85 participants [10] and in a multinational trial including 263,525 patients [11], rehospitalisation rates due to heart failure were 32.2% or 19 per 100 patient-years in the Empagliflozin group and (30.8%) or eight per 100 patient-years [10] and 13.6 events per 100 patient-years [11]. In other studies, the mean rate of hospitalisation was 2.91 per person-year in the first year after advanced HF diagnosis [4]. Meta-analysis data show that the pooled estimate of one-year readmission was 35.7% [12].

In our trial, 10 (55.6%) of patients were not hospitalised within one year with heart failure decompensation. Seven (38.9%) of patients were hospitalised one to two times, and only 1 (5.5 %) patient was hospitalised more than two times with congestion. Compared with the literature data, the patient group in our trial had decreased rehospitalisation rates due to heart failure decompensation. Due to the uncontrolled design of the study, improvements could be achieved by adding vericiguat to treatment and by improving the tolerance of other guideline-directed therapies.

In our trial, 50% of patients experienced hypotension at admission, which limited the optimisation of medical therapy. Compared with the literature, 21.8% of heart failure patients had hypotension [13]. Owing to hypotension, patients received suboptimal doses of medication, which in the long term resulted in worse outcomes and more rehospitalisations (Figure 1). After the addition of vericiguat, patients' blood pressure tends to increase, allowing the doses of guideline-recommended medications to be optimised and improving patient prognosis. As the literature shows, hypotension while hospitalised for acute decompensated heart failure is an independent risk factor for adverse 30-day outcomes [13]; therefore, improving haemodynamics is an important part of optimising medical therapy, and initiating guideline-recommended quadruple therapy and up-titrating medication to optimal doses is important [5]. Vericiguat therapy was well tolerated, and all patients tolerated vericiguat at the optimal dose of 10 mg. Vericiguat initiation has no or little hypotensive effect, allowing it to be initiated after the cessation of intravenous diuretics.

The VICTORIA echocardiographic substudy revealed an increase in the left ventricular ejection fraction from 33.0 ± 9.4% to 36.1 ± 10.2%; p < 0.01 in the vericiguat group but similarly in the placebo group [14]. In our trial, the left ventricular fraction increased significantly on average from 27% to 33% (p=0.049). The improvements in both groups highlight the importance of combining optimal medical therapy with optimal medication doses, which can affect patients' health and long-term outcomes. Vericiguat is an adjuvant medication that increases the efficacy of existing treatments. Similarly, as in the published data [15] in our trial, NT-proBNP significantly decreased with optimal medical treatment that included vericiguat. The average decrease was 44.7% compared with the index admission. In the Victoria trial, similar findings showing a decrease in NT-proBNP levels were reported [16].

Current study findings align with previous publications showing that adding vericiguat to guideline standard heart failure treatment for patients with heart failure with reduced ejection fraction decreases rehospitalisation rates and improves left ventricular ejection fraction and decreases levels of NT-proBNP.

Study limitations

On the basis of the 2021 European Society of Cardiology (ESC) Guidelines for the diagnosis and treatment of acute and chronic heart failure, vericiguat may be considered, in addition to standard therapy for heart failure with a reduced ejection fraction, to reduce the risk of cardiovascular mortality and hospitalisations for heart failure, but the use of new drugs in daily practice is still limited; In our trial we present initial data of 18 patients which decreases opportunity to evaluate novel medication impact to bigger patient groups. Still getting statistically significant changes shows good potential for additional therapy.

Due to the design of the study, a lack of a control group is another limiting factor that should be improved in further studies. Because of small group of patient's selection bias could be present.

Continuing research and finding new ways to improve heart failure patients' quality of life, reducing rehospitalisation rates are important, so adding to the experience of real-life patients helps to advocate implementing new medications in clinical practice.

## Conclusions

However, small uncontrolled cohort limits the opportunity to directly compare outcomes with published literature; it still provides hypothesis-generating findings. Our study revealed that patients receiving optimal heart failure medical therapy, including vericiguat, have lower rehospitalisation rates than those reported in the literature for patients receiving standard care. Vericiguat in this trial was well tolerated; all patients were able to receive the recommended dose, and it shows clinical and laboratory improvements in real-life scenarios. Improving haemodynamics allows further titration of recommended quadruple therapy, which is correlated with better symptom control and improvement in quality of life. Observed hemodynamic and therapeutic optimisation effects require confirmation in larger prospective trials. Sharing clinical experience is important to promote novel practice implementation in regular patient care, as well as collecting practical multicentre data adds to global real-world evidence, highlighting the need for controlled prospective trials. 
